# Systematic Modification and Evaluation of Enzyme-Loaded Chitosan Nanoparticles

**DOI:** 10.3390/ijms22157987

**Published:** 2021-07-26

**Authors:** Paulo R. Lino, João Leandro, Lara Figueiredo, Mariana P. Amaro, Lídia M. D. Gonçalves, Paula Leandro, António J. Almeida

**Affiliations:** Research Institute for Medicines (iMed.ULisboa), Faculty of Pharmacy, Universidade de Lisboa, Av. Prof. Gama Pinto, 1649-003 Lisboa, Portugal; proquelino@ff.ulisboa.pt (P.R.L.); jptleandro@gmail.com (J.L.); lara.figueiredo@outlook.com (L.F.); m.amaro@campus.fct.unl.pt (M.P.A.); lgoncalves@ff.ulisboa.pt (L.M.D.G.)

**Keywords:** chitosan, hyaluronic acid, cyclodextrins, biocompatibility modulation, enzyme delivery, complement system, haemocompatibility, phenylketonuria, human phenylalanine hydroxylase, nanoparticles

## Abstract

Polymeric-based nano drug delivery systems have been widely exploited to overcome protein instability during formulation. Presently, a diverse range of polymeric agents can be used, among which polysaccharides, such as chitosan (CS), hyaluronic acid (HA) and cyclodextrins (CDs), are included. Due to its unique biological and physicochemical properties, CS is one of the most used polysaccharides for development of protein delivery systems. However, CS has been described as potentially immunogenic. By envisaging a biosafe cytocompatible and haemocompatible profile, this paper reports the systematic development of a delivery system based on CS and derived with HA and CDs to nanoencapsulate the model human phenylalanine hydroxylase (hPAH) through ionotropic gelation with tripolyphosphate (TPP), while maintaining protein stability and enzyme activity. By merging the combined set of biopolymers, we were able to effectively entrap hPAH within CS nanoparticles with improvements in hPAH stability and the maintenance of functional activity, while simultaneously achieving strict control of the formulation process. Detailed characterization of the developed nanoparticulate systems showed that the lead formulations were internalized by hepatocytes (HepG2 cell line), did not reveal cell toxicity and presented a safe haemocompatible profile.

## 1. Introduction

The use of nano-sized drug delivery systems (DDS) to improve the pharmacokinetic and pharmacodynamic profile of biopharmaceuticals has attained a paramount role within current biomedical research. Nevertheless, due to an usual intricate labile nature, the delivery of complex biomacromolecules, such as peptides and proteins, is still considered a major challenge [1]. Although therapeutic proteins possess greater potency and specificity than other synthetic molecules, their beneficial potential may, however, be hindered by the loss of function during pharmaceutical manipulation and storage due to protein instability, limited bioavailability and putative immunogenicity [2]. In this context, enzyme packaged in a delivery vehicle possessed several advantages over soluble formulations that help to overcome protein instability and potential immunogenicity while also offering the possibility of higher targeting capabilities. In recent years, biopolymeric-based nanoparticles (NP) have proved not only to be safe, but also effective systems for the formulation and delivery of enzyme-therapeutics. Generally regarded as biocompatible and biodegradable, polysaccharides, such as chitosan (CS), hyaluronic acid (HA) and cyclodextrins (CDs), are among the most utilized and studied biopolymeric-based DDS (Figure S1) [3]. The linear glycosaminoglycan HA is one of the major constituents of the human extracellular matrix being ubiquitously distributed in almost all body fluids and tissues. The HA is a charged polymer (Figure S1) that has been extensively applied in several biomedical applications such as viscosupplementation in osteoarthritis, viscoelastic in eye surgery, wound regeneration and dermal filler [4]. Cyclodextrins have also received widespread attention within complex drug delivery for their cyclic cone-shaped structure with a hydrophobic core and a general hydrophilic surface (Figure S1), a property that has been exploited for their ability to complex with poorly soluble drugs and, thus, improving bioavailability and stability [5]. The linear cationic polysaccharide CS is composed of d-glucosamine and a varying number of *N*-acetyl-d-glucosamine residues, which determines its deacetylation degree (DD) (Figure S1). With a large number of hydroxyl groups, CS presents extremely flexible hydrodynamic properties and favourable chemical reactivity to interact with negatively charged labile drugs. Chitosan allows the use of mild methods to entrap the target proteins and offers the advantage of allowing the structural manipulation of the NP system for optimization of size, shape, rigidity, surface charge and chemistry [6], which are parameters that are described to influence the NP pharmacokinetics [7], cellular entry mechanism and intracellular trafficking patterns [8]. In addition to a diverse set of biomedical applications (e.g., DDS, tissue engineering, bioimaging, etc.) CS itself also present a diverse set of biological properties such as immunoenhancing, antifungal, antimicrobial and antioxidant activities [9].

Recently, we reported the rational design of a DDS based on the ionic gelation of CS with tripolyphosphate (TPP) for the formulation of the labile 200 kDa homotetrameric human phenylalanine hydroxylase (hPAH), which is a structurally and functionally complex enzyme [10]. Impaired hPAH activity, due to mutations in the encoding gene, is responsible for phenylketonuria (PKU), which is the most common genetic disorder of amino acid metabolism [11]. The optimized mild and self-controlled system allowed the encapsulation of hPAH in CS nanoparticles, maintaining the enzyme’s functional and biophysical properties. However, CS has been reported as potentially immunogenic and it has also been generally described as a haemostatic material that by a complex series of events, such as protein adsorption, platelet and leukocyte adhesion and activation of coagulation pathway, may potentially result in thrombogenicity [12]. In line with these observations, depending on its DD, molecular mass (MM) and protonation, CS has been applied as an adjuvant in the development of vaccines and as a haemostatic and complement activating material. Nevertheless, several authors have proven that careful manipulation and modification of its nano-bio interface and physicochemical properties may render CS nanoparticulate systems viable [13].

In recent years, in silico approaches have proved to be a relevant tool on the study of protein nanoformulation not only because it allows accessing the impact of nanomaterials on the amount and surface chemistry of the loaded biopharmaceutical [14], which can influences the stability of the formulated protein and interactions with biological components and cellular uptake, but also as it allows targeted modifications of protein residues in order to improve the biopharmaceutical characteristics [15]. Herein, combining both in silico and experimental methods, we present the development of a systematic matrix modification of the previously described CS-based system by using HAs with different MM, namely HA-50 (MM 20–50 kDa), HA-300 (MM 100–300 kDa) and HA-1000 (800–1000 kDa), as well as CDs with a different number of units and substituted with different moieties including β-cyclodextrin (β-CD), γ-cyclodextrin (γ-CD), carboxymethyl-β-cyclodextrin (CM-β-CD) and hydroxypropyl-β-cyclodextrin (HP-β-CD) with a degree of substitution (DS) of 0.65 (HP-β-CD 0.65) and 0.99 (HP-β-CD 0.9). The rationale was to further improve the stability of the complex 200 kDa hPAH, while being able to preserve enzymatic activity and to modulate/improve the system’s nano-bio interface with haematological and cellular compatibility, while maintaining a robust flexibility towards possible future administration routes.

## 2. Results and Discussion

### 2.1. CS-TPP Matrix Modification with Hyaluronic Acid 

**Figure 1:**
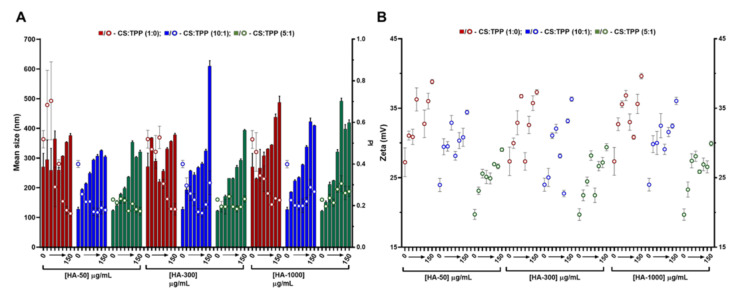
Characteristics of the chitosan-tripolyphosphate (CS-TPP) nanoparticles modified with hyaluronic acid (HA). Different HA loadings (0, 4, 8, 13, 17, 42, 83 and 150 μg/mL), HA molecular mass (HA-50, HA-300 and HA-1000) and CS:TPP ratio (1:0; 10:1 and 5:1) were assessed. (**A**) Mean size (bars) and PI (symbols) at CS:TPP mass ratio of 1:0 (red), 10:1 (blue) and 5:1 (green). (**B**) Zeta potential at CS:TPP mass ratio of 1:0 (red), 10:1 (blue) and 5:1 (green). Values represent mean ± S.D. (*n* = 3).

The hydroxyl-rich cationic nature of CS has previously been applied to nanoencapsulate the hPAH protein. At optimized conditions, CS with a DD of 91.8% and a MM of 61 kDa presented an improved polymeric conformation at an initial pH formulation of 6.0. Within these parameters, the main mechanistic bottleneck that impacted both colloidal self-assembly and enzyme functionality was the balance between the negative charges added by TPP and hPAH, as discussed elsewhere [16]. A modification of the CS matrix with a biocompatible anionic polysaccharide (such as HA) could improve hPAH stability [17,18]. In addition, HA, which is one of the main constituents of the extracellular matrix, could confer a stealth-like nano-bio interface with the intended host tissue depending on the possible route of administration. Starting from the previously optimized conditions (initial CS at 1 mg/mL and pH of 6.0), a systematic approach was designed to modify the CS-TPP empty NP with HA. By varying the CS:TPP ratio (1:0; 10:1 and 5:1), HA MM (50, 300 and 1000 kDa) and HA concentrations (4, 8, 13, 17, 42, 83 and 150 μg/mL), the nano-sized system’s design space was assessed up to colloidal destabilization and visible aggregation. The higher concentrations of HA, depending on its MM (HA-50: 417, 833, 1250 and 1500 μg/mL; HA-300 and HA-1000: 417, 833 μg/mL) were also tested (data shown in Figure S2). Within each tested HA MM, the polymeric self-assembly behaved in a similar manner with the addition of increasing amounts of TPP, as HA was added to the matrix, which improves the control of the ionic gelation of CS with NP with lower mean sizes and polydispersity index (PI) (Figure 1A).

As the TPP increased, the averages of the NP mean sizes demonstrated a decreasing tendency for HA-50 (from 310 ± 40 to 232 ± 72 nm), HA-300 (from 309 ± 50 to 231 ± 66 nm) and HA-1000 (from 334 ± 66 to 292 ± 115 nm) (Table S1). The same trend was observed for PI where the average values tended to decrease for HA-50 (from 0.39 ± 0.18 to 0.20 ± 0.02), HA-300 (from 0.36 ± 0.13 to 0.21 ± 0.02) and HA-1000 (from 0.32 ± 0.09 to 0.25 ± 0.03). At each HA concentration, the addition of TPP contributed to the neutralization of CS amines, resulting in a general decrease in zeta potential (Figure 1B). In fact, as the TPP concentration increased, the calculated zeta potential average decreased for HA-50 (from 33 ± 3.2 to 25 ± 1.9 mV), HA-300 (from 32 ± 3.2 to 25 ± 2.8 mV) and HA-1000 (from 34 ± 2.9 to 26 ± 2.3 mV). 

In the absence of TPP (CS:TPP molecular mass of 1:0), although higher NP average sizes are present, the addition of negative charges exclusively from HA allowed for an equalization of the CS:HA mass ratio of 1:1 (both at the final concentration of 833 µg/mL; Figure S2). In the case of the lowest MM HA (HA-50), even a polymeric system inversion is reached (HA:CS) at the maximum payloads of 1250 and 1500 µg/mL (Figure S2). Taking into account the substantial increase in MM from HA-50 to HA-1000 and that for HA-300 and HA-1000 and a MM of the modifying HA agent being higher than the CS base polymer (61 kDa), the impact on the colloidal system correlated positively with the higher intrinsic viscosity and bulkier nature of the increased MM HA.

We recently described how at an optimized initial pH of 6.0, CS acquires a helical conformation that favoured a more controlled ionic gelation [10]. Interestingly, the increasing amounts of each HA in the absence of TPP (Figure 1; CS:TPP mass ratio of 1:0) followed a similar behaviour to the previously reported TPP loading. From an initially destabilized system at small amounts of added negative charges, an optimized controlled formulation equilibrium was found with narrower size distribution (Figure 1A), after which destabilization/aggregation occurred once more. In both cases, the equilibrium was preceded by a zeta potential minimum which occurred at 17 (HA-300) and 42 µg/mL (HA-50 and HA-1000) of HA (50:1 and 20:1 CS:HA mass ratio, respectively) that resulted from a conformational shift of the nearly neutral packed helical CS polymeric chains identifying the molecular conditions from which a controlled ionic gelation occurred [16]. Such effect was balanced with the addition of the previously optimized TPP negative charges as it occurred at lower TPP amounts in the bare CS-TPP NP. More importantly and within controlled formulation conditions, for each MM HA an effective modulation of NP size and matrix modification dependent on HA concentration was achieved. 

Given the above results, to further assess the impact of HA on the system’s biocompatible profile, a lower loading and higher loading of 4 and 13 µg/mL HA (CS:HA 200:1 and 66:1 mass ratios, respectively) at a CS:TPP mass ratio of 5:1 were selected to proceed the study. Under such conditions, the NPs presented nano-sized favourable features, as the mean sizes for the tested MM HA were ≈150 (4 μg/mL HA) and 220 nm (13 μg/mL).

### 2.2. CS-TPP Matrix Modification with Cyclodextrins

**Figure 2:**
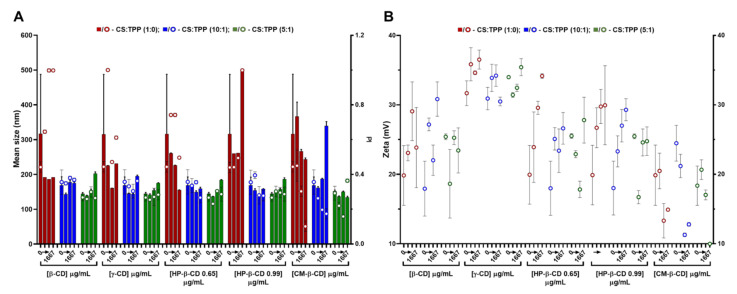
Characteristics of the chitosan.tripolyphosphate (CS-TPP) nanoparticles modified with cyclodextrins (CDs). Different CDs loadings (0 417, 833 and 1667 μg/mL), CD derivatives (β-CD, γ-CD, HP-β-CD 0.65, HP-β-CD 0.99 and CM-β-CD) and CS:TPP ratio (1:0; 10:1 and 5:1) were assessed. (**A**) Mean size (bars) and PI (symbols) at CS:TPP mass ratio of 1:0 (red), 10:1 (blue) and 5:1 (green). (**B**) Zeta potential at CS:TPP mass ratio of 1:0 (red), 10:1 (blue) and 5:1 (green). Values represent mean ± S.D. (*n* = 3).

Regarding CDs, these cyclic polysaccharides have been extensively applied to the complexation and physicochemical stabilization of complex drugs and inclusion in polysaccharide-based nanocarriers [5]. In the present study, CDs with a different number of glucose units, namely β-CD (seven) and γ-CD (eight); as well as different β-CD derivatives, namely CM-β-CD (with a DS of 3) and HP-β-CD (with a DS of 0.65 and 0.99) (Figure S1) were screened. After a preliminary assessment (data not shown), a variable CD loading was implemented at previous TPP and CS optimized conditions. Compared to HA, the lower CDs MM (ranging from 1.14 kDa for β-CD up to 2.35 kDa for CM-β-CD) and neutral nature (except for CM-β-CD) had less impact in the colloid’s physical properties (Figure 2). Thus, system modification with CDs was boosted towards higher CS:CD mass ratios, namely 2:1, 1:1 and 1:2 (417, 833 and 1667 µg/mL CD). Again, increasing the CS:TPP ratio (1:0, 10:1 and 5:1) improved the polymeric self-assembly and produces more compact NPs with a narrower size distribution (Figure 2A and Table S1). At the optimal CS:TPP 5:1 mass ratio, with an initial CS pH of 6.0, the inclusion of neutral CDs (β, γ and HP-β) resulted in a controlled dose-dependent modulation of NP size (from ~140 to 180 nm), while maintaining a controlled narrow size distribution (PI ~0.25) up to the system’s inversion at the highest CD amount of 1667 µg/mL (1:2; CS:CD mass ratio) (Figure 2A). 

Such behaviour did not significantly impact on the system’s surface charge (Figure 2B). As for the anionic CM-β-CD, the ionic gelation resembled CS self-assembly more closely with HA (Figure 1). In this case, the increasing amount of negative charges from CM-β-CD was able to improve the control in the colloidal system even in absence of TPP, (decreasing PI up to ~0.1; Figure 2A). With a DS of three and taking into account that at the final formulation pH of 6.5 the carboxyl moieties are negatively charged, each CM-β-CD is actually contributing with 21 negative charges (opposed to the TPP triple negative charge at pH of 6.5) [10]. Moreover, particularly at the optimized CS:TPP mass ratio of 5:1, the addition of CM-β-CD maintained the NP mean size at lower values, with a decreased surface charge. 

The previous optimization of CS NP regarding concentration and protonation status proved to be critical in the formulation process [10]. With similar CS to CD ratios, other authors were only able to produce NP from HP-β-CD with a mean size ≥300 nm and a PI ~0.3 and, from CM-β-CD, a mean size ≥230 nm was obtained [19]. Thus, having obtained controlled conditions for every CD tested, formulations at CS:TPP mass ratio of 5:1 and at a lower and higher loading of CD, namely 417 and 833 µg/mL (2:1 and 1:1 CS:HA mass ratio), were selected to proceed to the cytocompatibility and haemocompatibility assays.

### 2.3. Cyto and Haemocompatibility of the Modified CS-TPP Matrix

Nanoparticulate tailoring aimed at the modulation of the interface between a colloidal system and a given biological host has been extensively sought [20,21]. Aiming at the intravenous or subcutaneous route of administration, cellular and haemocompatibility screenings were performed with the selected array of empty NP optimized systems. 

#### 2.3.1. Cytotoxicity and Nanoparticle Uptake

**Figure 3:**
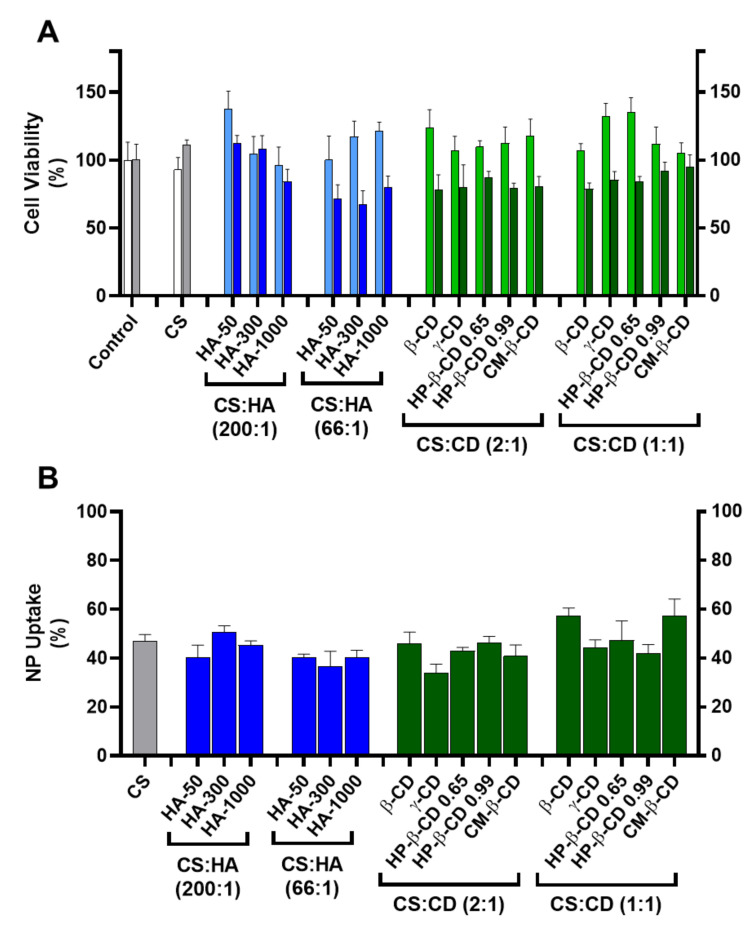
Cellular toxicity and uptake of the chitosan-tripolyphosphate (CS-TPP) nanoparticles modified with hyaluronic acid (HA) and cyclodextrins (CDs). The CS-TPP matrix (CS:TPP at 5:1 mass ratio) was modified with a low (CS:HA 200:1) and high loading (CS:HA 66:1) of HA with different molecular mass (HA-50, HA-300 and HA-1000) and different CD (β-CD, γ-CD, HP-β-CD 0.65, HP-β-CD 0.99 and CM-β-CD) at a low (CS:CD 2:1) and high loading (CS:CD 1:1). (**A**). Cellular viability on HEK293T (light blue and light green) and HepG2 (dark blue and dark green) cell lines; (Control) culture media with no added nanoparticles in HEK293T (white) and HepG2 (grey) cell lines; (CS) (CS) CS-TPP non modified nanoparticles in HEK293T (white) and HepG2 (grey) cell lines. (**B**). Uptake profile in HepG2 cell lines monitored by fluorescence analysis with Oregon green stained nanoparticles; (CS) CS-TPP non modified nanoparticles (grey). Values represent mean ± S.D. (*n* = 3).

As a first biocompatibility screen a cell viability assay was implemented using HEK293T (human embryonic kidney epithelial cell line, ATCC CRL-11268) and HepG2 (human hepatocellular carcinoma cell line, ATCC HB-8065) cell lines (Figure 3A). 

Incubation of HEK293T cells with every tested formulation at a final concentration of 83.3 µg/mL did not show a significant cellular toxicity than when compared to the CS:TPP formulation (93 ± 9%) where cell viability ranged from 138 ± 13% (for CS:HA-50 at 200:1 mass ratio) to 96 ± 13% (for CS:HA-1000 at 200:1 mass ratio). Regarding HepG2, this cell line proved to be more sensitive to modified NP (Figure 3A). For the CS-TPP NP, an uptake of ~47% after 24 h of incubation was observed with no impact in cell viability (111 ± 3%). At the lower HA loading (CS:HA mass ratio of 200:1), inclusion of lower MM HA (HA-50 and HA-300) did not impact cellular viability (112 ± 6% and 108 ± 10%, respectively). However, the inclusion of HA-1000 induced a slight decrease in Hep G2 viability down to 84 ± 9%; Figure 3A). At the higher HA loading of 66:1 (CS:HA mass ratio), the impact of the HA MM was dissipated and cell viability of the three modified empty NP formulations was significantly hampered (from 67 ± 10% to 80 ± 8%). Such behaviour has been attributed to the CD44 receptor and extensively described as a key modulator of the proliferation and growth inhibition of certain human tumours (such as the hepatoma HepG2 cells) by regulation of their cell–cell and cell–matrix adhesion pathways (primarily linked to HA) [22]. However, this same interaction did not apparently confer any vectorization properties towards the hepatocytes with respect to NP uptake and when compared to the CS:TPP NP (47 ± 3%) at the lower HA loading (CS:HA mass ratio of 200:1) there was no significant increase in NP uptake (37 ± 6 and 51 ± 3% for HA-300 at 66:1 and 200:1 mass ratios, respectively; Figure 3B). Regarding the CDs modified systems there was also a general slight impact in HepG2 viability, which ranged from 78 ± 4% (CS:β-CD at 1:1 mass ratio) to 95 ± 9% (CS:CM-β-CD at 1:1 mass ratio; Figure 3A). The observed behaviour may result from the CD complexation with cellular membrane cholesterol and its solubilisation which has been vastly studied as a cytotoxicity inducing effect [23]. Interestingly, regarding the NP uptake, at the lower CD loading (CS:CD mass ratio of 2:1), except for γ-CD (34 ± 3%) there were no significant deviations over the CS-TPP system (47 ± 3%). Moreover, at the higher CD loading (CS:CD mass ratio of 1:1) an increase in NP uptake occurred with β-CD (57 ± 3%) and CM-β-CD (57 ± 7%) while the remaining formulations maintained the profile of the bare system (Figure 3B). 

#### 2.3.2. Haemocompatibility

As NPs come into contact with a biological fluid such as blood and the complete array of its circulating components, a protein interfacial ensemble surrounds the delivery system (protein corona) and usually dictates its biodistribution, incidence of adverse events, therapeutic potency and removal by the mononuclear phagocytic system [24,25,26]. As CS has been described as a haemostatic material [12], a careful evaluation of the haemocompatibility profile of modified CS NP was performed in this work by analysing haemolysis degree, platelet, coagulation and complement activation [25].

**Figure 4:**
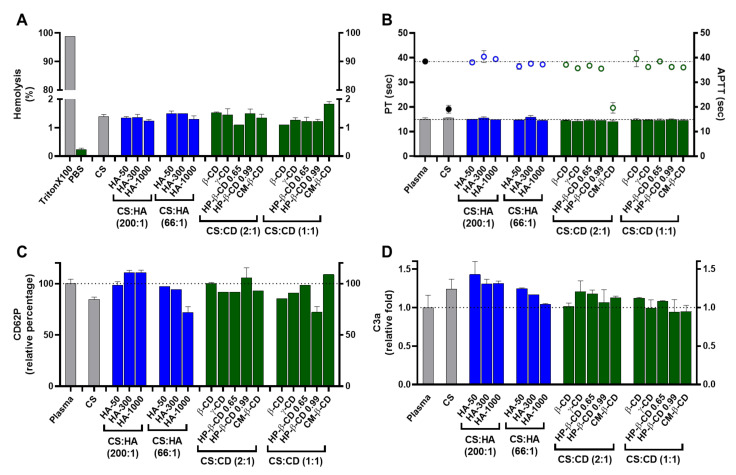
Haemocompatibility profile of the chitosan.tripolyphosphate (CS-TPP) nanoparticles modified with hyaluronic acid (HA) and cyclodextrins (CDs). The CS-TPP matrix (CS:TPP at 5:1 mass ratio) was modified with low (CS:HA 200:1) and high loading (CS:HA 66:1) of HA with different molecular masses (HA-50, HA-300 and HA-1000) and different CD (β-CD, γ-CD, HP-β-CD 0.65, HP-β-CD 0.99 and CM-β-CD) at high (CS:CD 2:1) and low loading (CS:CD 1:1). (**A**) Haemolysis percentage monitored by the cyanomethaemoglobin method after nanoparticle incubation in whole blood; (**B**) Coagulation assays of PT (bars) and APTT (symbols) after nanoparticle incubation in plasma; (**C**) Platelet activation monitored by the release of CD62P after nanoparticle incubation in platelet-rich plasma; (**D**) Complement activation monitored by the release of C3a desArg fragment after nanoparticle incubation in plasma. (CS) CS-TPP non-modified nanoparticles. Values represent mean ± S.D. (*n* = 3). See text for details. In (**B**–**D**) the lines represent the reference values of control plasma.

According to the American Society for Testing and Materials (ASTM) E2524-08 standard criterion, materials are classified as non-haemolytic (haemolysis <2%), slightly haemolytic (haemolysis 2–5%) and haemolytic (haemolysis > 5%) [27]. As shown in Figure 4A, the tested nanoformulations presented haemolysis percentages of ~1.3% irrespective of the modifying agent and loading ratio except for the highest loading of CM-β-CD (~1.8%) and thus can be classified as non-haemolytic. According to Nadesh et al. [28], this modifies the dispersion medium of CS from acetic acid to saline. By modifying the NP surrounding pH, these authors significantly improved the haemolysis profile of CS NP and still obtained ~20% of haemolysis. As the degree of CS amine protonation and the surface charge are key modulators of the NP interaction with blood components, our previously optimized formulation pH strongly improved this haematological compatibility endpoint. The neutralization of CS has also been described as critical in the interaction with other types of cells, including prokaryotes where the ionic status of CS amines is crucial for the development of antimicrobial activity [29].

Regarding thrombogenesis, the tested NP did not induce a visible clot formation. In addition, platelet activation was monitored by the release of CD62P (Figure 4C). Platelet activation is considered relevant when an increase of >20% in the release of CD62P (when compared to the control) is obtained [30]. As shown in Figure 4C, the tested NP did not promote platelet activation as the percentage of CD62P release relative to control was always <20%, ranging from 72 ± 5.5% (HA-1000 at CS:HA 66:1 mass ratio) to ≈111 ±2.1% (HA-300 and HA-1000 at CS:HA 200:1 mass ratio). Moreover, the CS-TPP system and the formulations with HA-1000 and HP-β-CD 0.99 at the highest loading induced a slight decrease in CD62P release which has been attributed to the unspecific protein adsorption at the NP protein corona. A similar effect occurred for the C3, C5 and factor B complement proteins, which were adsorbed to CS NP without cleavage or activation of their specific immune pathways [31].

Concerning plasma coagulation, this complex process is controlled by the activation of either the intrinsic or extrinsic pathways and can also be a major drawback in NP design. Cascade activation or inhibition may confer a risk of thrombogenesis or haemorrhage, respectively. Both intrinsic and extrinsic pathways were monitored by the APTT (activated partial thromboplastin time) and the prothrombin and PT (prothrombin time) assays, respectively (Figure 4B). APTT and PT variations are considered significant for |values| > 5% when compared to the control assay [32]. As observed in Figure 4B, the CS-TPP system and NP modified with the lowest loading of CM-β-CD did change the intrinsic pathway as their APTT were ≈−50% of the control. The remaining formulations presented no significant variation towards the control, proving how an efficient modulation of the NP matrix is able to regulate their interaction with the blood components and avoid the procoagulant activity generally attributed to CS (Figure 4B) [12]. For the PT assay, no significant variations towards the control were observed for the tested formulations (Figure 4B). 

Elements of the complement system are among the several circulating proteins that may interact with NP, constituting the major constituents of a delivery system’s protein corona [33]. The complement system plays a major role in the opsonization and immune recognition of foreign materials, thus affecting the biodistribution properties of a colloidal carrier and impacting its safety and biocompatibility profile by inducing the recognition of the mononuclear phagocytic system and potentiating both a rapid clearance from circulation and possible immunogenic adverse events [34]. The complement system is composed of three major pathways (classical, alternative and lectin pathways) and a broad number of components. Nevertheless, the activation of all pathways results in the cleavage of the C3 component and the release of C3a, which is thus utilized to monitor for complement activation. Values are considered relevant when released C3a are more than 2-fold higher than control assay [35]. As shown in Figure 4D for the tested NP, the C3a release was always ≤1.4-fold higher than the control. Nevertheless, there were some differences in the C3a released levels that correlated with the different polymeric system modifications. The CS-TPP system presents a mild but statistically significant increase in the released C3a towards the control (1.2-fold, *p* = 0.0168; Figure 4D). Several studies have demonstrated that some CS-based systems (films and NP) possess the ability to activate the complement system but consensus is lacking as to how such process occurs and how the modulation and/or dissipation of this immune reaction may be implemented [12]. The process of opsonization is described as mainly dependent from random Brownian motions, but when NP comes into contact with complement components, any of several attractive forces including van der Walls, electrostatic, ionic, hydrophobic/hydrophilic and others can be involved in the binding and activation of opsonins at their surface [34]. Thus, the observed mild increase is a direct consequence of the previous optimized ionic balance between CS and TPP, which rendered CS amines at the final formulation pH (close to neutralization) less reactive [10].

Regarding the modification with HA, a decrease in C3a release as HA MM and loading ratio increased was observed. In fact, HA-50 at its lowest loading presents the highest C3a release (1.4-fold compared to control; Figure 4D). As the MM and HA loading increases, C3a release decreases to values similar to the control, which suggests an improved immune modulation over the CS-TPP system. In vivo fragmentation of HA ( ≥1000 kDa) usually occurs because of tissue damage and oxidative stress. This results in a pro-inflammatory response, which is mainly attributed to the interaction of generated HA fragments and the immune CD44 receptor [36]. From this perspective, the increased molecular hindrance of higher HA MM and loading ratios will confer an improved dose-dependent modulation of the system’s immune response up to an apparent stealth-like status.

For the CD modified NP, interesting results were also observed. A C3a release similar to that caused by the CS bare system was found only for the γ-CD at the lowest loading (Figure 4D). For the remaining conditions, the complexation of CDs within the designed polymeric CS matrix resulted in an effective dissipation of complement activation through the protection of the CS moieties chemically prone to interact with this set of proteins. Moreover, CD have also been described as being able to modulate the activation and cellular recognition of C3 through the extraction of membrane cholesterol and perturbation of lipid rafts (which are essential in immune signal transduction mechanisms) [37].

### 2.4. hPAH Nanoencapsulation in Selected CS Modified Systems

We previously assessed how hPAH impacted the physicochemical and functional properties of CS-TPP NP by shifting the enzyme’s molecular crowding and solvent exposure profile [10]. For that system, the best compromise between the protein encapsulation efficiency (EE), enzymatic activity and thermal stability was obtained at a hPAH final concentration of 100 µg/mL. In order to further assess the derived system’s compatibility and to probe the conditions with increased protein stabilization potential and improved putative therapeutic potency, the hPAH loading capacity was increased until a protein final concentration of 250 µg/mL was attained (CS:hPAH of 3:1 mass ratio).

**Figure 5:**
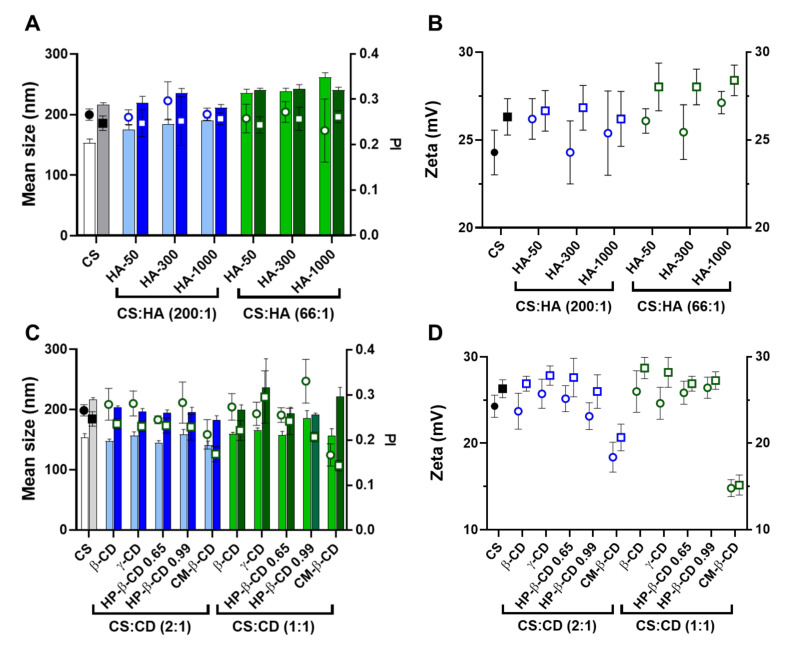
Comparative analysis of the characteristics of empty and human phenylalanine hydroxylase (hPAH) loaded chitosan-tripolyphosphate (CS-TPP) nanoparticles modified with hyaluronic acid (HA) and cyclodextrins (CDs). (**A**) Mean size (bars) and PI (symbols) of empty (light blue and light green) and hPAH loaded nanoparticles (dark blue and dark green) modified with HA of different molecular mass (HA-50, HA-300 and HA-1000); (**B**) Zeta potential of empty (black, blue and green circles) and hPAH loaded nanoparticles (black, blue and green squares) modified with HA of different molecular mass (HA-50, HA-300 and HA-1000); (**C**) Mean size (bars) and PI (symbols) of empty (light blue and light green) and hPAH loaded nanoparticles (dark blue and dark green) modified with different CDs derivatives; (**D**) Zeta potential of empty (black, blue and green circles) and hPAH loaded nanoparticles (black, blue and green squares) modified with different CDs derivatives. NPs were obtained using 5:1 CS-TPP and CS:HA-300 at 200:1 mass ratio; CS:HP-β-CD 0.99 and CS:CM-β-CD at 2:1 mass ratio; hPAH encapsulation at a final concentration of 250 µg/mL.Values represent mean ± S.D. (*n* = 3).

Concerning the NP physicochemical properties, the inclusion of a high load of hPAH resulted in a general increase in the mean NP size from the bare system (Figure 5A,C), while maintaining a controlled PI (~0.25). 

The optimized mild formulation method and modifying agents permitted retaining or the slight increase in NP superficial charge (Figure 5B,D). We previously showed how hPAH is mildly negatively charged at the final formulating pH of ~6.5 [10]. A zeta potential decrease points to an hPAH mainly entrapped within the interior of the NP matrix without extensive solvent exposure. A slight increase in superficial charge occurs as additional negative charges are added from hPAH inducing the collapse of more positively charged CS chains into the colloidal system (increased size and zeta potential; Figure 5B,D). 

By taking a closer look into the HA modified systems, the slight increase in mean NP size with the increment of both HA’s MM and concentration observed for the empty NP is dissipated with the inclusion of the enzyme (Figure 5A). This is explained by the pre-incubation of hPAH with TPP and HA, where the enzyme’s high load competes with the TPP and HA negative charges for the ionic gelation of CS amines, resulting in more homogeneous NP sizes throughout the tested conditions (~220 nm). 

Regarding CDs, apart from the specific case of γ-CD and CM-β-CD, the general impact in the colloidal system was more limited. Interestingly, when compared to the hPAH loaded CS:TPP NP (216 ± 3 nm), the mean size of the CD-modified formulations was slightly decreased for the 2:1 ratio formulations (~195 nm; *p* value ranging 0.0231 to <0.0001) (Figure 5C). Considering that hPAH is previously incubated with higher amounts of CDs, a protection of hPAH negatively charged residues by CD complexation is evidenced when compared to HA. The complexation of CM-β-CD with hPAH considerably improved the control (PI ~0.15) of the ionic gelation, which is more evident for the 1:1 ratio formulations (Figure 5C,D). Likewise, CM-β-CD negative charges also induced a decrease in the NP superficial charge (Figure 5D).

### 2.5. Encapsulation Efficiency, Enzyme Activity and Thermal Stability of hPAH Loaded Nanoparticles

In order to evaluate and compare the activity of hPAH loaded NP, enzyme activity was always determined as specific activity (nmol l-Tyr/min/mg protein). Under standard conditions, the activity of the “naked” hPAH was 4430 ± 351 nmol l-Tyr/min/mg. This value was considered as 100% to obtain the relative enzyme activity. 

**Figure 6:**
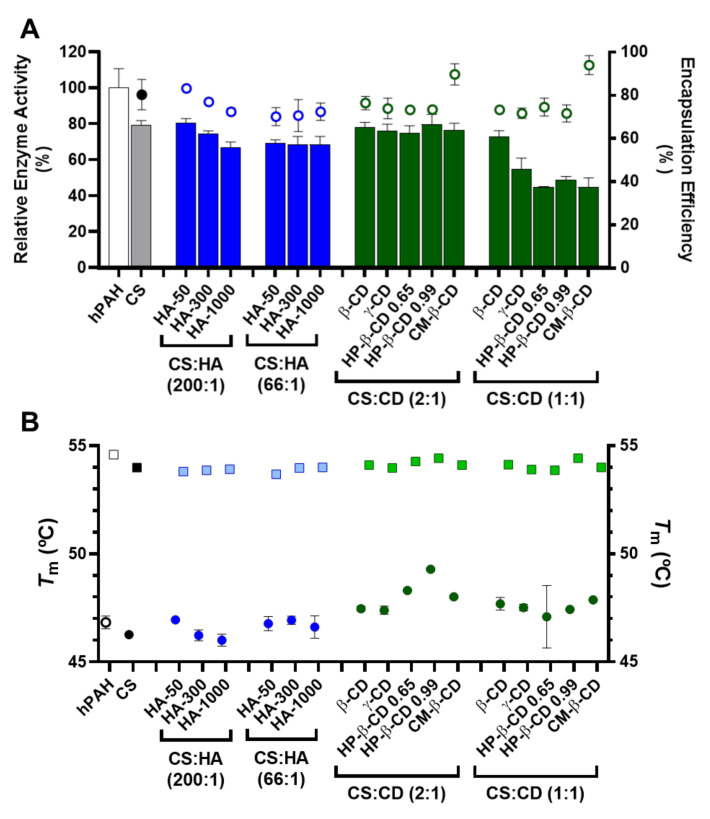
Encapsulation efficiency (EE), enzyme activity and thermal stability of human phenylalanine hydroxylase (hPAH) encapsulated in chitosan.tripolyphosphate (CS-TPP) nanoparticles modified with hyaluronic acid (HA) and cyclodextrins (CDs). (**A**) Relative enzyme activity (bars) determined at standard conditions (1 mM l-Phe, 75 µM BH_4_, 25 °C; bars) and encapsulation efficiency (EE; symbols) monitored by the BCA protein assay. (**B**) Thermal stability of the regulatory (round symbols; T_m1_) and catalytic (square symbols; T_m2_) domains of buffered and encapsulated hPAH. NPs were obtained using 5:1 CS-TPP and CS:HA-300 at 200:1 mass ratio; CS:HP-β-CD 0.99 and CS:CM-β-CD at 2:1 mass ratio; hPAH encapsulation at a final concentration of 250 µg/mL. Values represent mean ± S.D. (*n* = 3).

**Figure 7:**
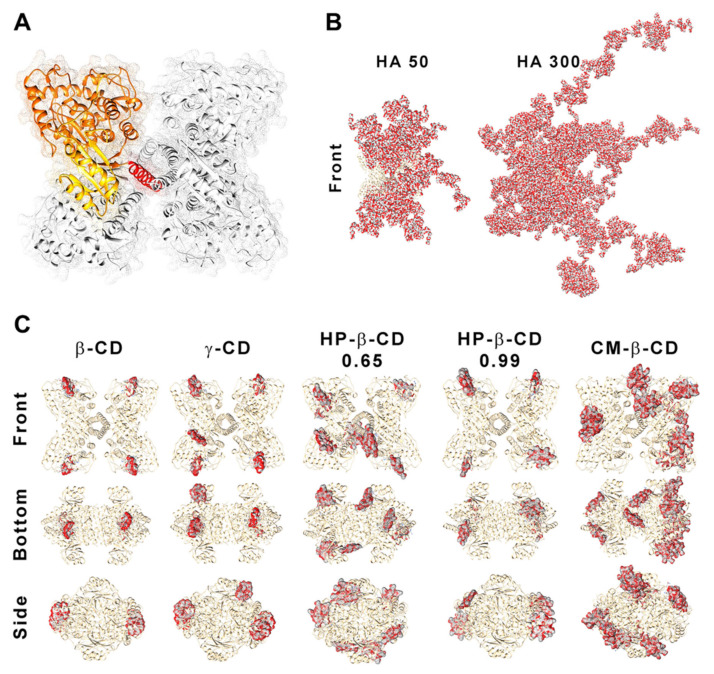
Structure of human phenylalanine hydroxylase (hPAH) and its interaction with hyaluronic acid (HA) and cyclodextrins (CDs). (**A**) Homotetrameric quaternary structure of hPAH with the regulatory (yellow), catalytic (orange) and oligomerization (red) domains of a monomer evidenced. Depiction of the interaction/complexation of hPAH with HA-50 and HA-300 (**B**) and with CDs derivatives (**C**). Structures were obtained by superposition of the 10 best blind docking hits with generated HA polymeric chains and CDs structures.

According to our data, the competition between hPAH and HA for the ionic gelation of CS amines was further evidenced by the significant decrease in both protein EE and enzyme activity with the increase in HA MM at the lowest CS:HA mass ratio of 200:1 (from 80 to 70%; Figure 6A). At CS:HA of 66:1 mass ratio, the HA MM impact on the two parameters above was dissipated. The stability of encapsulated protein was monitored using differential scanning fluorimetry (DSF) by determining the hPAH melting temperatures (*T*_m_) from the midpoint of the first (*T*_m1_) and second (*T*_m2_) transitions. Interestingly, the above suggested interaction did not impact the thermal stability of nanoencapsulated protein where DSF was able to show a preserved hPAH *T*_m1_ and *T*_m2_ (Figure 6B), which has been associated to the unfolding of the regulatory and catalytic domains, respectively [38]. The blind in silico molecular docking (Figure 7B) suggests that the unspecific molecular hindrance of an increasing MM of HA impacts both hPAH interactions with CS amines (decreased EE) and enzyme functionality through the access to catalytic substrates (decreased enzymatic activity; Figure 6A). For the CD modified NP, at the smaller protein loading of CS:CD at 2:1 mass ratio and except for CM-β-CD, the impact on protein EE and enzyme activity was negligible when compared to the CS bare system (Figure 6A). Nevertheless, although functionality was preserved, the preincubation and complexation of hPAH with CD (particularly HP-β-CD and CM-β-CD) induced thermal protein stabilization, particularly of the regulatory domain (*T*_m1_) (Figure 6B). 

We previously showed how this transition, which is associated with the less stable and aggregation-prone regulatory domain, positively shifted as hPAH was nanoencapsulated and the balance between CS and hPAH was modified [10]. When compared to the *T*_m1_ of free hPAH (46.8 ± 0.3 °C) and hPAH-CS loaded NP (46.2 ± 0.07 °C), incubation with CD resulted in an increase in *T*_m1_ from 47.1 ± 1.4 °C (HP-β-CD 0.65 at 1:1 mass ratios) to 49.3 ± 0.06 °C (HP-β-CD 0.99 at 2:1 mass ratios). By increasing the hPAH loading within the NP system from the previously described optimum final concentration of 100 µg/mL [10] to 250 µg/mL, we were able to test the system response under stress conditions. Stabilizing conditions were found that allowed the improvement of the viability of higher hPAH:CS ratios with consequently higher putative therapeutic potency. Interestingly, such effect is also conditioned by the molecular structure of the complexed cyclic polysaccharides. The low increase in the CD MM from β-CD to γ-CD (≈1.14 to 1.30 kDa) did not significantly impact hPAH thermal stability (*p* = 0.9076; Figure 6B). Nevertheless, with the HP-β-CD, as the DS increased (0.65 to 0.99 hydroxypropyl moieties per glucose unit) an increase in the stabilization of the regulatory domain also occurred. For the specific CM-β-CD, the CD additional negative charges induced a significant increase in EE at the smaller loading of CS:CD at a mass ratio of 2:1 once more (from ~80 to 90%; *p* = 0.0073; Figure 6B). Still, no visible impact on hPAH functionality was observed. 

Blind molecular docking revealed how CD tends to interact with hPAH (Figure 7C and Figure S3). The best model of every CD shows specific complexation with the regulatory hPAH domain. Establishment of intermolecular hydrogen bonds and chemical contacts with this region will probably stabilize this domain and, thus, induces an increase in *T*_m1_ (higher thermal stability) (Figure 6B). At the larger CS:CD mass ratio of 1:1, although no significant impact occurred regarding protein EE, the relative enzymatic activity generally dropped down to ~45% (Figure 6A). As hPAH thermal stability was maintained, the enzymatic functional decrease correlates with an excess of free CD available to complex with the enzymatic substrate and cofactor (added during the enzymatic activity assay).

### 2.6. Nano-Bio Interface with Selected hPAH-Loaded Nanoparticles

**Figure 8:**
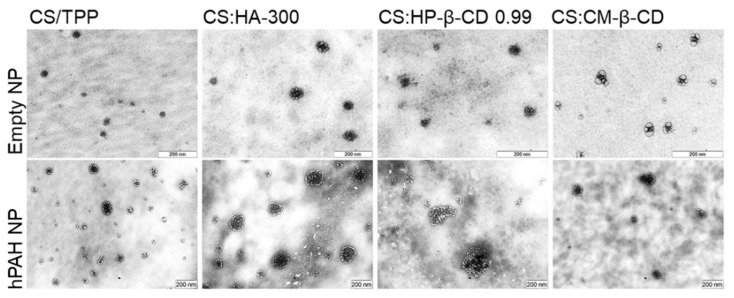
Morphological analysis by transmission electron microscopy (TEM) of empty and hPAH loaded chitosan/tripolyphosphate (CS/TPP) nanoparticles (NP) modified with hyaluronic acid (HA) and cyclodextrin (CDs) derivatives. NPs were obtained using 5:1 CS-TPP and CS:HA-300 at 200:1 mass ratio; CS:HP-β-CD 0.99 and CS:CM-β-CD at 2:1 mass ratio; hPAH encapsulation at a final concentration of 250 µg/mL. See text for details.

In view of these results, a selected group of hPAH-containing formulations was used to proceed to the biocompatibility studies. Hence, in addition to the buffered protein and the CS-TPP NP, the systems modified with HA-300 (200:1 mass ratio of CS:HA), HP-β-CD 0.99 and CM-β-CD (both at 2:1 mass ratio of CS:CD) were selected as the best compromise between the identified critical parameters. The TEM (transmission electron microscopy) analysis of the selected systems confirmed the light scattering measurements shows a spherical homogeneous nanoparticle population with similar mean particle sizes either in the presence or absence of hPAH (Figure 8).

**Figure 9:**
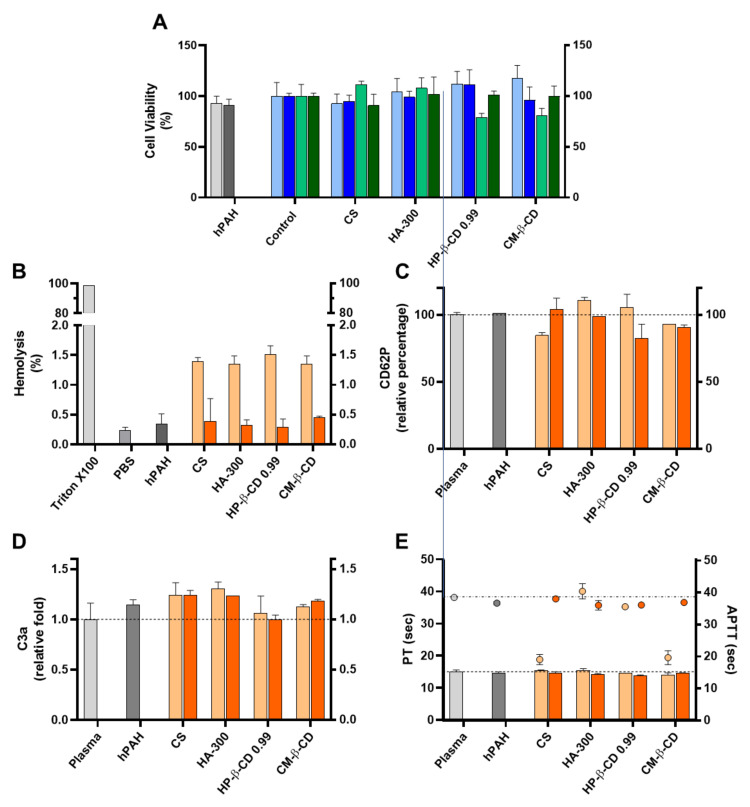
Assessment of the cellular toxicity and haemo and immune nano-bio interface of human phenylalanine hydroxylase (hPAH) chitosan/tripolyphosphate (CS/TPP) nanoparticles (NP) modified with hyaluronic acid (HA) and cyclodextrin (CDs) derivatives. (**A**) Cellular viability of HEK293T (light and dark blue) and HepG2 (light and dark green) upon incubation with empty (light blue and light green) and hPAH loaded (dark blue and dark green) NP; Control refers to the cells assays performed only with culture medium. (**B**) Haemolysis degree monitored by the cyanomethaemoglobin method after incubation of empty (light orange) and hPAH-loaded (dark orange) NP in whole blood; TritonX100 represents total haemolysis and PBS buffer in the negative control assay; (**C**) Platelet activation monitored by the release of CD62P incubation of empty (light orange) and hPAH-loaded (dark orange) NP in platelet-rich plasma. (**D**) Complement activation monitored by the release of C3a desArg fragment after incubation of empty (light orange) and hPAH-loaded (dark orange) NP in plasma; (**E**) Coagulation assays of PT (bars) and APTT (symbols) after incubation of empty (light orange) and hPAH-loaded (dark orange) NP in plasma. In (**C**,**D**), plasma was used as the control. In all assays the naked hPAH (hPAH) was also used as a control. NPs were obtained using 5:1 CS-TPP and CS:HA-300 at 200:1 mass ratio; CS:HP-β-CD 0.99 and CS:CM-β-CD at 2:1 mass ratio; hPAH encapsulation at a final concentration of 250 µg/mL. Values represent mean ± S.D. (*n* = 3).

For biocompatibility studies, the inclusion of hPAH effectively improved the results obtained for the CS empty systems. As previously described, every HA and CD was incubated and/or complexed with hPAH before the ionic gelation, having significant impacts both on the colloidal physicochemical properties and the recovered enzyme’s functional and stability behaviour. An improved biosafe profile was obtained for every tested parameter, in particular, for the viability assays in HepG2 cells, haemolysis degree and APTT (Figure 9).

The further optimized neutralization of CS amines with hPAH and its complexion with the excipients (particularly CD), dissipated the cellular viability issues in the HepG2 cell lines (Figure 9A). Interestingly, as shown in Figure 9B, the mild and negligible haemolysis observed for the empty NP (from 1.4 to 0.3%) was further reduced in the presence of hPAH-loaded NP ranging from 0.28 ± 0.14% (CS:HP-β-CD 0.99) to 0.45 ±0.03% (CS:CM-β-CD), approaching the value obtained in the presence of PBS (0.23 ± 0.06%). The haemostatic activation of APTT followed the same trend (Figure 9E) as in the presence of hPAH-loaded CS and hPAH-loaded CS:CM-β-CD the APTT increased from 19.1 ± 1.6 sec. to 38.0 ± 0.2 sec. and from 19.6 ± 2.1 sec. to 36.9 ± 0.01 sec., respectively (plasma control 38.5 ± 0.8 sec.). For the remaining parameters (CD62p and C3a release and PT), no strong effects were observed, although for the CS:HA-300 and CS: HP-β-CD 0.99, a slight decrease in CD62P release (Figure 9C), C3a release (Figure 9D) and PT (Figure 9E) could be observed when compared to the empty NP. 

## 3. Materials and Methods

### 3.1. Materials and Chemicals

*Escherichia coli* TOP 10, the prokaryotic expression vector pTrcHis and the fluorescent dye SYPRO orange (5000× stock concentration) were obtained from Invitrogen (Carlsbad, CA, USA). The cofactor tetrahydrobiopterin (BH_4_), catalase, l-Phenylalanine (l-Phe), Hepes, dithiothreitol (DTT), low MM CS (61 kDa; viscosity: 42 cps) with a DD of 91.8%, TPP, cibacron brilliant red 3B-A and CM-β-CD with a DS of 3 were from Sigma Chemical Co (St. Louis, MO, USA). Hydrolysed HA ranging MM of 20–50 (PrimaHyal 50; HA-50), 100–300 (PrimaHyal 300; HA-300) and 800–1000 kDa (PrimaHyal 1000; HA-1000) were from Soliance (Pomacle, France). The β-CD (Cavamax W7) and γ-CD (Cavamax W8) were from Wacker Chemie AG (Munich, Germany). The HP-β-CD with DS of 0.65 (HP-β-CD 0.65; Kleptose HPB) and 0.99 (HP-β-CD 0.99; Kleptose HP) were from Roquette Corporate (Lestrem, France). Glycerol (molecular biology grade) was purchased from Merck KGaA (Darmstadt, Germany). All reagents were of analytical grade or equivalent.

### 3.2. Expression and Purification of Recombinant hPAH Tetramers

The hPAH recombinant protein was expressed in *E. coli* as a fusion protein as previously described [39] upon isopropyl-β-d-thiogalactoside (IPTG; 1 mM) induction for 3 h at 37 °C. The soluble fraction was used to purify the fusion protein. Recombinant proteins were purified by immobilized metal affinity chromatography using a Ni-chelating resin (Qiagen, Valencia, CA, USA) and eluted with 250 mM imidazole in a sodium phosphate buffer, as previously reported [40].

Size exclusion chromatography (SEC) was further used to isolate recombinant hPAH tetramers in a 1.6 cm × 60 cm HiLoad Superdex 200 HR column (GE-Healthcare Life Sciences; Uppsala, Sweden) and with a mobile phase containing 20 mM Na-Hepes, 200 mM NaCl, pH 7.0 (SEC buffer), pumped at a flow rate of 0.7 mL/min [41]. All purification steps were carried out at 4 °C. The tetrameric fusion protein was concentrated using an Amicon Ultra 15 centrifugal filter (MWCO 30 kDa; Millipore; Billerica, MA, USA). The concentration of purified tetramers was determined by considering that a solution of hPAH at 1 mg/mL/cm has an absorbance of 0.91 at λ280 nm (absorption coefficient *A*_280_). 

### 3.3. Preparation of CS Nanoparticles

The particulate system was obtained by ionic gelation using the previously optimized method described elsewhere [10]. Briefly, stock solutions of CS at 10 mg/mL in 1% (*v*/*v*) acetic acid, TPP and CDs at 10 mg/mL in ultra-pure water and HA at 1 or 0.1 mg/mL in ultra-pure water were prepared. The stock solutions of TPP and the remaining modifying agents were diluted in SEC buffer to the desired concentrations. The pH of CS solution at 1 mg/mL was increased to 6.0 and NPs were instantly formed when TPP/modifying agent (CDs or HA) solution was added to CS at a previously optimized 1:5 fixed volume ratio (CS at 833 μg/mL) [10]. Starting from previously optimized conditions, the system was modified by varying the TPP concentration, modifying agent concentration and the presence of the protein. In this case, the preparation of protein loaded NP was performed using isolated hPAH tetramers and previously incubated with TPP/modifying agent (at a concentration suitable to maintain the volume ratio and final desired concentrations). Non-encapsulated protein was removed by centrifugation of the NP at 40,000× *g* for 30 min at 4 °C.

### 3.4. Particle Size, Distribution, Zeta Potential and Encapsulation Efficiency

Mean particle size and PI were determined by photon correlation spectroscopy on a Zetasizer Nano-S (Malvern Instruments; Malvern, Worcestershire, UK). Zeta potential was measured by using laser Doppler anemometry on a Zetasizer Nano-Z (Malvern Instruments). Samples were diluted with 0.45 µm filtered ultra-pure water. In all cases, mean values were obtained from the analysis of three different batches and each of them was measured three times. 

In order to determine the EE, the amount of encapsulated protein was determined indirectly by the BCA protein assay (Thermo Fisher Scientific, Rockford, IL, USA) and the following equation was used:

(1) $$\mathrm{EE}\;\left(\%\right)=\frac{{\left[\mathrm{hPAH}\right]}_{\mathrm{total}}-{\left[\mathrm{hPAH}\right]}_{\sup}}{{\left[\mathrm{hPAH}\right]}_{\mathrm{total}}}\times 100$$

where [hPAH]_total_ represents the total amount of hPAH added to each sample and [hPAH]_sup_ the amount of hPAH present in the supernatant after NP deposition. 

The absence of proteolytic degradation before and after encapsulation was monitored by polyacrylamide gel electrophoresis under denaturing conditions (SDS-PAGE, 10% gel) using Coomassie Brilliant Blue R250 staining (data not shown).

### 3.5. Transmission Electron Microscopy (TEM)

The morphology of NP was examined by TEM on a H-8100 microscope (Hitachi High-Technologies Corporation, Tokyo, Japan). Nanoparticles were deposited in 200 mesh Cu formvar/carbon support film grids (Pelco^®^ Tem grid Support Films, Ted Pella, Inc., Redding, CA, USA), dried for 10 min and then further analysed.

### 3.6. Enzymatic Activity of hPAH Loaded Nanoparticles 

The hPAH activity was measured as previously described [42]. Briefly, the reaction mixture in a 200 μL final volume contained 1 mM l-Phe, 0.1 M Na-Hepes, pH 7.0, 0.1 mg/mL catalase, 5 μg of recombinant hPAH tetramers, 5 mM dithiothreitol DTT and 100 μM ferrous ammonium sulphate. After pre-incubation with l-Phe, the reaction was started by the addition of 75 μM BH_4_. The amount of l-Tyr produced after 1 min was quantitated by HPLC using a LiChroCART^®^ 250-4 LiChrospher^®^ 60 RP-select B (5 μm) column (Merck KGaA), a 5% ethanol mobile phase pumped at 0.7 mL/min flow rate and fluorimetric detection (λ_exc_= 274 nm and λ_em_= 304 nm) [41]. Enzyme activity was expressed as nmol of l-Tyr formed during one minute per milligram of hPAH (nmol Tyr/min/mg). 

### 3.7. Differential Scanning Fluorimetry

Thermal unfolding profiles of hPAH were obtained by DSF, in a C1000 Touch thermal cycler with a CFX96 optical reaction module (Bio-Rad; Hercules, CA, USA) as described in [10]. All assays were carried out in SEC buffer and with Sypro Orange at a 2.5× final concentration. The PCR plates were sealed with Optical-Quality Sealing Tape (Bio-Rad) and centrifuged at 500× *g* for 5 min. The thermal profiles were obtained by ramping the temperature between 20 and 90 °C at 1 °C/min, with a 1 s hold time every 0.2 °C and fluorescence acquisition through the FRET channel. Data were analysed with CFX Manager Software V3.0 (Bio-Rad) and GraphPad Prism software V6.00 (La Jolla, CA, USA), fitting the experimental curves with a biphasic dose–response function to obtain the hPAH *T*_ms_ from the midpoint of the first (*T*_m1_) and second (*T*_m2_) transitions.

### 3.8. In Silico Modelling

The 3D representations of CDs and HA polymeric chains were generated with Materials Studio 7.0 (Accelrys, Inc, San Diego, CA, USA) according to their described MM and DS. For HA, a small oligomer of two d-glucoronic acid monomers intercalated with two *N*-acetyl-d-glucosamine linked via alternating β-1,4 and β-1,3 glycosidic bonds was generated and its structure further refined with the steepest descend energy minimization and compass forcefield. The oligomer was then replicated 43, 247 and 1111 times to reproduce the theoretical average MM of HA-50 (35 kDa), HA-300 (200 kDa) and HA-1000 (900 kDa). CDs were also designed in silico according to their DS and further refined with the steepest descend method. In order to analyse the impact of these molecules on hPAH structure, a composite full-length model was generated from the truncated forms of rat PAH (PDB ID: 1PHZ) and hPAH (PDB ID: 2PAH) by using the UCSF Chimera (University of California, San Francisco, CA, USA) [43], as described in [10]. The full-length composite PQR file was charged at the final formulation pH (6.5) with PROPKA and PDB2PQR using the optimized forcefield for Poisson–Boltzmann calculations PEOEPB and further submitted to blind molecular docking with the analysed HAs and CDs in the PatchDock server as described in [10]. For each blind docking assay the best 1000 hits were refined with FireDock server and sorted according to the minimum global energy (attractive/repulsive van der Waals, atomic contact energy and hydrogen bonding) [44]. Docking with HA-1000 was not performed due to the large number of atoms of the generated polymeric chain that surpassed the limit of atoms able to be inserted in a PDB file. The 10 hits scoring the lowest global energy were overlapped in the rendered figures and an elementary surface depiction was created with the solvent-excluded molecular surfaces MSMS package [45].

### 3.9. Cell Viability

The NP cytotoxicity was assessed in HEK293T and HepG2 cells using the MTT (3-(4,5-dimethyl-2-thiazolyl)-2,5-diphenyl-2H-tetrazolium bromide) cell viability assay [46]. Briefly, the cells were seeded in 96-well tissue culture plates in RPMI 1640 culture medium (Gibco Life Technologies, Carlsbad, CA, USA) supplemented with 10% foetal serum bovine, 100 units/mL of penicillin G (sodium salt), 100 μg/mL of streptomycin sulphate and 2 mM l-glutamine at a cell density of 2 × 10^4^ cells per well. Cells were incubated at 37 °C and 5% CO_2_ in a humidified atmosphere with 10 µL of empty or hPAH loaded NP (83 µg/mL final concentration) and the non-encapsulated protein and culture medium used as controls. After 24 h incubation, cell media was removed and replaced with fresh medium containing MTT dye at 0.5 mg/mL. After 3 h of incubation, media were removed and the intracellular formazan crystals were solubilized and extracted with dimethyl sulfoxide. After 15 min at room temperature, the absorbance was measured at λ570 nm on a Microplate Reader FLUOstar Omega (BMG Labtech GmbH, Ortenberg, Germany). The relative cell viability was determined using the following equation:

(2) $$\mathrm{Cell}\;\mathrm{viability}\;\left(\%\right)=\frac{{\mathrm{Abs}}_{\mathrm{test}}}{{\mathrm{Abs}}_{\mathrm{control}}}\times 100$$

where Abs_test_ is the absorbance value obtained for sample treated cells and Abs_control_ the absorbance value obtained for cells incubated with culture medium.

### 3.10. Assessment of the Nanoparticles Uptake Profile

In order to evaluate the profile of the cellular uptake, NP were labelled with the Oregon Green^®^ 488 (Life Technologies, Carlsbad, CA, USA) probe by following the manufacturer’s instructions. Briefly, the NP solution was incubated with Oregon Green^®^ 488 for 1 h, at 37 °C, in the dark with gently stirring. Excess Oregon green was further removed by SEC using a Sephadex G-200 (GE-Healthcare Life Sciences) equilibrated with 20 mM Hepes buffer at pH 7.4. The labelled NP were eluted in SEC buffer and further used.

HepG2 cells were grown as described above for the in vitro cell viability assays. The culture medium was replaced by 100 µL of medium containing 20 µL of Oregon Green labelled NP and cells were further incubated for 24 h at 37 °C and 5% CO_2_ in humidified atmosphere. After the 24 h incubation period, cells were washed three times with 250 µL of pre-warmed (37 °C) PBS buffer containing 20 mM glycine at pH 7.4. The washing solution was removed and cells were disrupted with 100 µL 1% Triton ×100 solution. Fluorescence was measured at λ_exc_485 nm and λ_em_520 nm on a Microplate Reader FLUOstar Omega. Internalization was calculated as a percentage considering the fluorescence obtained immediately after the labelled NP addition as 100%. 

### 3.11. Haemocompatibility Assays

Whole blood was obtained from healthy volunteer donors after institutional ethical approval and appropriate informed consent. The blood was collected in EDTA or sodium citrate tubes and pooled before the assays. Blood compatibility tests were performed by evaluating haemolysis, platelet activation, plasma coagulation time and complement activation, as detailed below. Samples of test solution containing hPAH loaded-NP as well as empty NP, non-encapsulated protein and PBS buffer were used. Additionally, adequate controls were performed for each particular test. All the assays were performed in triplicate.

The percentage of haemolysis induced by NP was evaluated in EDTA-anticoagulated blood [47,48]. NP samples (833 µg/mL) were incubated with the pooled blood at a blood:sample ratio of 100:20 (*v*/*v*) and incubated at 37 °C for 2 h under mild shaking conditions. PBS-treated blood (spontaneous lysis) and Triton X-100 1% (*w*/*v*)-treated blood (100% lysis) were used as controls. Samples and control-treated blood were centrifuged at 800× *g* for 15 min at 25 °C. In order to estimate the extent of erythrocyte lysis, the released haemoglobin was measured in the obtained supernatant using the Drabkin reagent and colorimetric detection at 540 nm. The haemolytic property of nanoparticles was plotted as a percentage of haemolysis which was calculated according to the following equation:

(3) $$Haemolysis\;\left(\%\right)=\frac{\left[test\;Hb\right]}{\left[positive\;control\;Hb\right]}\times 100$$

where positive control haemoglobin concentration is the value of haemoglobin in the pooled whole blood (Triton X-100 treated sample) and test haemoglobin concentration is the haemoglobin released into plasma when the blood is exposed to the test sample.

Platelet activation by NP was evaluated by measuring the soluble P-selectin release using platelet-rich plasma (PRP), which was obtained by centrifugation of sodium citrate-anticoagulated blood at 200× *g* for 15 min at 22 °C [30]. NP samples (833 µg/mL) were incubated with the PRP at a ratio of 25:100 (*v*/*v*) at 37 °C for 15 min under mild shaking conditions. Untreated PRP was used as a negative control. The degree of platelet activation was measured using a CD62P (P-Selectin) Human ELISA Kit (ab100631 Abcam, Cambridge, UK) according to the manufacturer’s protocol.

The effect on plasma coagulation time was evaluated by determining the prothrombin time (PT) and the activated partial thromboplastin time (APTT) in blood collected in sodium-citrate vacutainers [32]. To this end, plasma obtained after blood centrifugation at 2500 rpm for 10 min at 22 °C was incubated with the NP samples (833 µg/mL) at a sample:plasma ratio of 30:270 (*v*/*v*) at 37 °C for 30 min, under mild shaking conditions. The PT and APTT were determined in a coagulation analyzer (Coatron M2; Teco, Munich, Germany) according to the manufacturer’s instructions. In order to assess complement activation, the cleavage of complement component C3 was monitored by measuring the formation of its activation peptide (C3a desArg) [48]. Blood collected in sodium-citrate vacutainers was centrifuged at 2500 rpm for 10 min at 22 °C. The obtained plasma was further incubated with the NP samples (833 µg/mL) at a ratio of 50:50 at 37 °C for 1 h, under mild shaking conditions. The C3a desArg concentration on assay samples was measured using the C3a enzyme ELISA kit (Sunred Biological Technology Co., Shanghai, China) according to the manufacturer’s instructions. The fold-increase in complement activation was calculated by comparing the concentration of C3a desArg in the test samples to the control baseline according to the following equation.

(4) $$Complement\;activation=\frac{\left[test\;C3a\;desArg\right]}{\left[control\;C3a\;desArg\right]}$$

### 3.12. Controls and Statistical Analysis

Adequate controls for the described assays consisted of the non-encapsulated hPAH tetramers in solutions containing the studied components at the tested concentrations (SEC buffer, TPP, CS or presence of modifying agent). Data from independent experiments (*n* ≥ 3) are shown as mean ± SD. When applied, statistical significance (*P*) was determined by the Student’s paired t-test and compared the hPAH with the corresponding control sample (*p* < 0.05 was considered significant).

## 4. Conclusions

Using a systematic design approach based on previously defined critical formulation parameters, we successfully modified a nanoparticulate carrier system with several HA and CDs. Having gained extensive insights into how each component of the colloidal system behaved, an optimum balance between the beneficial properties of system modification and protein loading resulted in an overall maintenance and/or improvement of the tested parameters. The selected functional nanoformulations are apparently suitable for different administration routes. By merging in silico, in vitro and in cellulo data, we arrived at comprehensive new insights into how free and enzyme-loaded colloidal systems may be systematically tailored toward a safe and effective nano-bio interface with a given tissue. The polyol-like nature of the nanobiomaterials may have been critical for improving enzyme stabilization as well as the tested biocompatibility of the developed formulations.

Furthermore, an in-depth knowledge of how these nanoparticulate polymeric systems behave and how cellular viability, haemostasis and the complement response may be activated or dissipated according to the therapeutic need at hand considerably improves their drug delivery potential. Within a regulatory setting, these results contribute to clarify some of the general nanotoxicological concerns that are perhaps wrongly attributed to an entire group of heterogeneous and flexible materials where CS is included. Each final colloidal system should be regarded with its own specificities. Hence, we provided a mechanistic development platform to formulate and deliver the complex hPAH and opened exciting new perspectives towards future formulations strategies for enzyme delivery towards potentially complex Enzyme Reposition Therapy addressable clinical presentations.

## Data Availability

Not applicable.

## Supplementary Materials

The following are available online at https://www.mdpi.com/article/10.3390/ijms22157987/s1, Figure S1: Chemical structure of raw materials utilized to obtained the nanoparticles, Figure S2: Characteristics of the chitosan.tripolyphosphate (CS-TPP) nanoparticles modified with hyaluronic acid (HA) at high HA loading of 417, 833, 1250 and 1500 μg/mL depending on HA molecular mass (HA-50, HA-300 and HA-1000) and using different CS:TPP ratios (1:0; 10:1 and 5:1), Figure S3: Depiction of the best blind molecular docking model of cyclodextrins (CDs; β-CD; γ-CD; HP-β-CD 0.65; HP-β-CD 0.99 and CM-β-CD) with human phenylalanine hydroxylase (hPAH) and their interaction with the protein regulatory domain, Table S1: Average values of mean size, polidispersity index (PI) and zeta potential of chitosan (CS) nanoparticles modified with hyaluronic acid (HA) of different molecular masses and cyclodextrins (CD) at different concentrations and using CS:tripolyphosphate (TPP) ratios of 1:0, 10:1 and 5:1.
