# Supplementary material for: Systematic Modification and Evaluation of Enzyme-Loaded Chitosan Nanoparticles
Source: Int J Mol Sci. 2021 Jul 26;22(15):7987. doi: 10.3390/ijms22157987 (PMC8348744; doi:10.3390/ijms22157987)
Supplement: Supplementary file 1 [file ijms-22-07987-s001.zip › ijms-1295413-supplementary.pdf]

International Journal of *Molecular Sciences*

SUPPLEMENTARY DATA

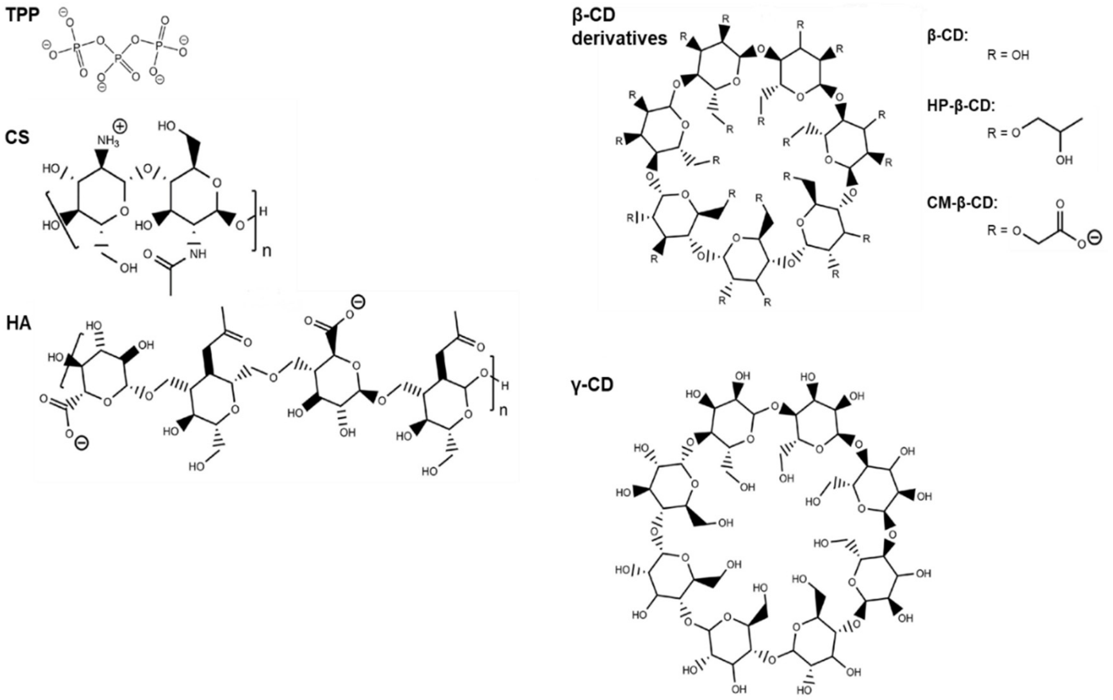

Figure S1. Chemical structure of raw materials utilized in nanoparticles. (TPP) tripolyphosphate; (CS) chitosan; (HA) hyaluronic acid; (CD) cyclodextrins: β-CD, hydroxypropyl- β-CD (HP-β-CD) carboxymethyl β-CD and γ-CD.

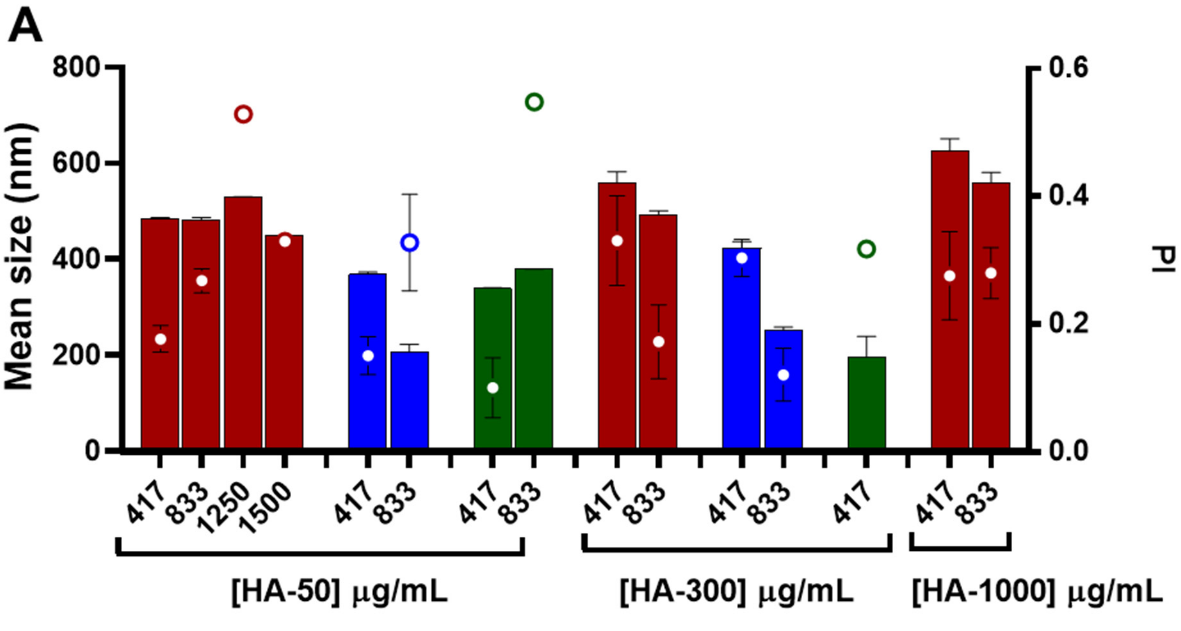

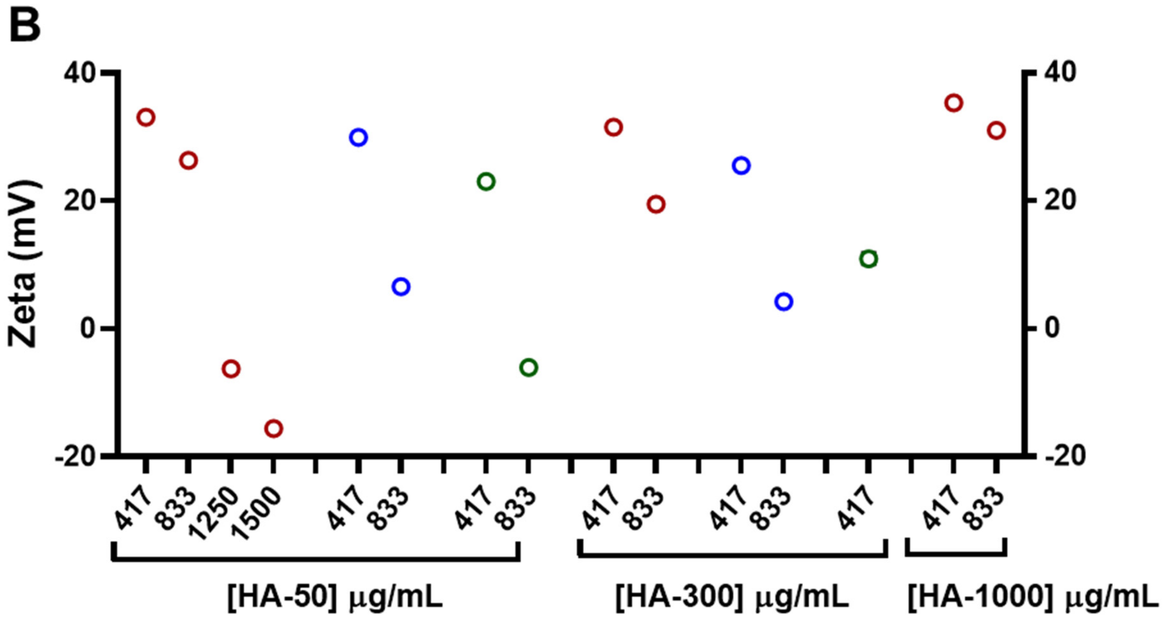

Figure S2. Characteristics of the chitosan-tripolyphosphate (CS-TPP) nanoparticles modified with hyaluronic acid (HA) at high HA loading of 417, 833, 1250 and 1500 μg/mL depending on HA molecular mass (HA-50, HA-300 and HA-1000) and using different CS:TPP ratios (1:0; 10:1 and 5:1). (A) Mean size (bars) and PI (symbols) at CS:TPP mass ratio of 1:0 (red), 10:1 (blue) and 5:1 (green). (B) Zeta potential at CS:TPP mass ratio of 1:0 (red), 10:1 (blue) and 5:1 (green). Values represent mean ± S.D. (n=3).

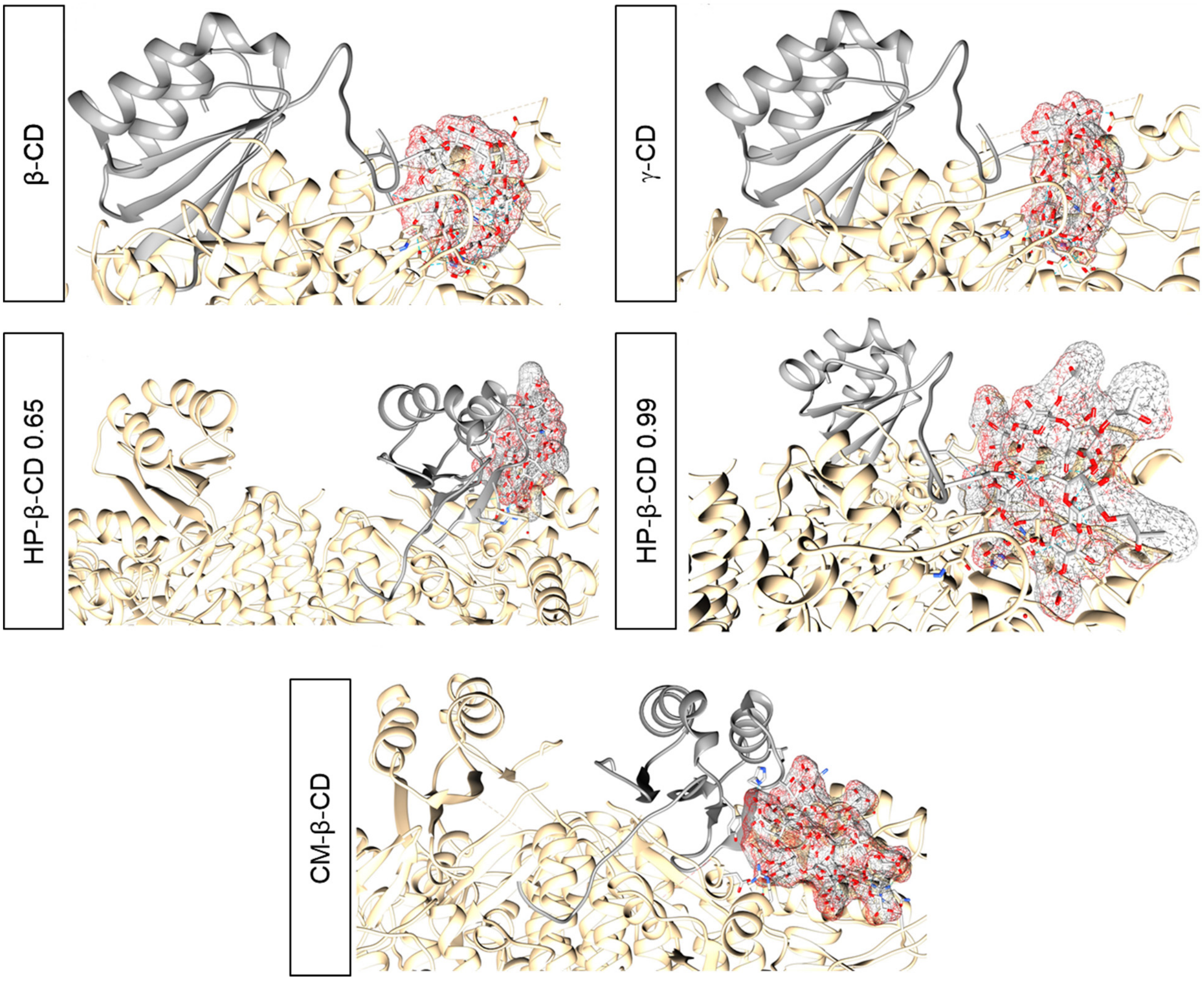

Figure S3. Depiction of the best blind molecular docking model of cyclodextrins (CDs; β-CD; γ-CD; HP-β-CD 0.65; HP-β-CD 0.99 and CM-β-CD) with human phenylalanine hydroxylase (hPAH) and their interaction with the protein regulatory domain. The hPAH regulatory domain is evidenced in grey; amino-acid residues interacting (hydrogen bonds and chemical contacts) with CDs are evidenced with the elemental colours and the specific hydrogen bonds are coloured in blue.

Table S1. Average values of mean size, polidispersity index (PI) and zeta potential of chitosan (CS) nanoparticles modified with hyaluronic acid (HA) of different molecular masses (HA-50; HA-300 and HA-1000) and different cyclodextrins (CD) derivatives (β-CD; γ-CD; HP-β-CD 0.65; HP-β-CD 0.99 and CM-β-CD) at different concentrations and using CS:tripolyphosphate (TPP) ratios of 1:0, 10:1 and 5:1. .

	CS:TPP Mass ratio	Mean Size (nm)	PI	Zeta Potential (mV)
HA-50	1:0	310 ± 40	0.39 ± 0.18	33 ± 3.2
	10:1	252 ± 56	0.22 ± 0.05	30 ± 2.2
	5:1	232 ± 72	0.20 ± 0.02	25 ± 1.9
HA-300	1:0	309 ± 50	0.36 ± 0.13	32 ± 3.2
	10:1	288 ± 89	0.25 ± 0.06	29 ± 4.1
	5:1	231 ± 66	0.21 ± 0.02	25 ± 2.8
HA-1000	1:0	334 ± 66	0.32 ± 0.09	34 ± 2.9
	10:1	277 ± 85	0.25 ± 0.05	31 ± 2.4
	5:1	292 ± 115	0.25 ± 0.03	26 ± 2.3
β-CD	1:0	222 ± 64	0.77 ± 0.28	24 ± 3.8
	10:1	166 ± 16	0.36 ± 0.01	24 ± 5.7

	5:1	160 ± 29	0.27 ± 0.02	23 ± 3.2
γ-CD	1:0	233 ± 64	0.63 ± 0.26	35 ± 2.1
	10:1	163 ± 154	0.34 ±0.03	32 ± 1.9
	5:1	154 ± 15	0.27 ± 0.01	33 ± 1.8
HP-β-CD 0.65	1:0	240 ± 68	0.60 ± 0.16	27 ± 6.2
	10:1	162 ± 10	0.33 ± 0.04	23 ± 3.8
	5:1	154 ± 21	0.27 ± 0.03	24 ± 4.3
HP-β-CD 0.99	1:0	333 ± 111	0.59 ± 0.27	26 ± 4.7
	10:1	154 ± 14	0.33 ± 0.06	24 ± 4.9
	5:1	159 ± 19	0.29 ± 0.02	23 ± 4.1
CM-β-CD	1:0	298 ± 55	0.32 ± 0.16	17 ± 3.6
	10:1	214 ± 84	0.25 ± 0.08	17 ± 6.4
	5:1	144 ± 9	0.26 ± 0.09	16 ± 5.4
